# ASO Author Reflections: Anastomotic Leaks After Esophagectomy—No Impact on Long-Term Survival

**DOI:** 10.1245/s10434-020-08212-3

**Published:** 2020-01-23

**Authors:** Sivesh K. Kamarajah, Alexander W. Phillips

**Affiliations:** 1 Northern Oesophagogastric Unit, Royal Victoria Infirmary, Newcastle upon Tyne NHS Foundation Trust, Newcastle-upon-Tyne, UK; 2 Institute of Cellular Medicine, Newcastle University, Newcastle-upon-Tyne, UK; 3 School of Medical Education, Newcastle University, Newcastle upon Tyne, UK

## Past

Anastomotic leak remains a common complication after esophagectomy. It is associated with perioperative mortality and prolonged hospital stay. Some have suggested that it may also contribute towards poorer long-term survival, although the mechanism is unclear. A French multicenter study defined severe esophageal leak (SEAL) as that which equated to a grade III/IV Clavien–Dindo complication. This study demonstrated a poorer long-term prognosis in patients who developed SEAL following esophagectomy;^1^ however, the study was limited by a very heterogenous population and did not report on recurrence-free survival. The question thus remains, does anastomotic leak negatively influence long-term survival in patients undergoing esophagectomy for cancer?

## Present

The present study evaluates outcomes from a single center over a 20-year period.^2^ Complications are recorded contemporaneously, as are patient outcomes. Oncological management decisions were made by a single multidisciplinary team, and surgical technique was standardized across those operating. This involved a radical two-field lymphadenectomy permitted by a transthoracic route.^3^ The consistency of these factors potentially increases the validity of these results in centers that have a similar surgical ethos. It may be that a radical lymphadenectomy,^4^ which was routinely performed within this study, negated any potential adverse effects on long-term prognosis. Reassuringly anastomotic leak, irrespective of severity, had no bearing on long-term survival. This is an important consideration for both patients and clinicians, allowing appropriate counselling and reassurance.

## Future

These data may help allay fears that anastomotic leak may potentially contribute towards poorer overall prognosis. The importance of managing leaks appropriately is vital for short-term outcomes. High-volume centers, by definition, have increased experience in managing such complications, and a judicious and aggressive strategy has been shown to lead to minimal risk of perioperative mortality from such leaks.^5^ However, techniques that might help prevent anastomotic leak remain a research priority. Concepts such as preconditioning and intraoperative use of indocyanine green to gauge conduit vascularity have all been employed but none have been able to eliminate the incidence of anastomotic leak. Further research into anastomotic techniques and adjuncts that can minimize leak occurrence or diminish their short-term impact are required.
